# Metabolome-Microbiome Responses of Growing Pigs Induced by Time-Restricted Feeding

**DOI:** 10.3389/fvets.2021.681202

**Published:** 2021-06-22

**Authors:** Hongyu Wang, Pengke Xia, Zhiyang Lu, Yong Su, Weiyun Zhu

**Affiliations:** ^1^Laboratory of Gastrointestinal Microbiology, Jiangsu Key Laboratory of Gastrointestinal Nutrition and Animal Health, College of Animal Science and Technology, Nanjing Agricultural University, Nanjing, China; ^2^National Center for International Research on Animal Gut Nutrition, Nanjing Agricultural University, Nanjing, China

**Keywords:** growing pigs, metabolome, microbiome, time-restricted feeding, microbial metabolites

## Abstract

Time-restricted feeding (TRF) mode is a potential strategy in improving the health and production of farm animals. However, the effect of TRF on microbiota and their metabolism in the large intestine of the host remains unclear. Therefore, the present study aimed to investigate the responses of microbiome and metabolome induced by TRF based on a growing-pig model. Twelve crossbred growing barrows were randomly allotted into two groups with six replicates (1 pig/pen), namely, the free-access feeding group (FA) and TRF group. Pigs in the FA group were fed free access while the TRF group were fed free access within a regular time three times per day at 07:00–08:00, 12:00–13:00, and 18:00–19:00, respectively. Results showed that the concentrations of NH4-N, putrescine, cadaverine, spermidine, spermine, total biogenic amines, isobutyrate, butyrate, isovalerate, total SCFA, and lactate were increased while the pH value in the colonic digesta and the concentration of acetate was decreased in the TRF group. The Shannon index was significantly increased in the TRF group; however, no significant effects were found in the Fisher index, Simpson index, ACE index, Chao1 index, and observed species between the two groups. In the TRF group, the relative abundances of *Prevotella 1* and *Eubacterium ruminantium group* were significantly increased while the relative abundances of *Clostridium sensu sticto 1, Lactobacillus*, and *Eubacterium coprostanoligenes group* were decreased compared with the FA group. PLS-DA analysis revealed an obvious and regular variation between the FA and TRF groups, further pathway enrichment analysis showed that these differential features were mainly enriched in pyrimidine metabolism, nicotinate and nicotinamide metabolism, glycerolipid metabolism, and fructose and mannose metabolism. In addition, Pearson's correlation analysis indicated that the changes in the microbial genera were correlated with the colonic metabolites. In conclusion, these results together indicated that although the overall microbial composition in the colon was not changed, TRF induced the gradient changes of the nutrients and metabolites which were correlated with certain microbial genera including *Lactobacillus, Eubacterium_ruminantium* group, *Eubacterium coprostanoligenes* group, *Prevotella* 1, and *Clostridium* sensu sticto 1. However, more studies are needed to understand the impacts of TRF on the health and metabolism of growing pigs.

## Introduction

Recently, a considerable amount of reference highlighted the contributions of the type, quantity, and composition of nutrients intake to host health and metabolism both on animals and human beings (1–4). By contrast, there are fewer studies concerning the effects of feeding patterns on health and metabolism. However, to date, limited literature has already indicated that changes in feeding patterns may also affect the metabolism, health, and production of animals (5, 6). Specifically, Rothschild et al. (7) reviewed that TRF trends to reduce the body weight, total cholesterol, and concentrations of triglycerides, glucose, insulin, interleukin, and tumor necrosis factor-α with improving insulin sensitivity (7). Consistently, TRF was reported to ameliorate the serum lipid and liver profiles of the individuals and increased the richness of the gut microbiota on human beings (8). Zarrinpar et al. (9) reported that compared with the FA group, TRF has changed the dynamics both in the relative abundance and compositions of gut microflora on mice as well as liver metabolism. These studies concerning mice and human beings together indicated that TRF has profound effects on the host metabolism and may be a potential remedy for the prevention of metabolic diseases and promoting a profitable and safe swine production. However, the mechanisms underpinning the beneficial effects of TRF on metabolic health remain largely unknown.

In recent years, more and more evidence has indicated that the microbiota plays a crucial role in host metabolism and health (10, 11). Vice versa, factors including diet composition (12), nutritional concentrations (13), and diet types (14) were found to shape the microbial communities. Therefore, we hypothesized that TRF changed the concentration gradient of nutrients, thus, preferentially stimulating the proliferation of certain microorganisms in the intestine and further exerting its beneficial effects on host health and metabolism. As obesity has become a global concern and has a close relationship with food taking, related studies mostly focus on indicators such as glucose and lipid metabolism, which are mostly performed on mice. More studies concerning the effects of TRF on gut microbiota and relevant metabolome are needed. Hence, the present study aimed to explore the metabolome-microbiome responses of growing pigs induced by a time-restricted feeding and to study the relationships between the gut microbiota, gut environment, and metabolites. Specifically, the feeding pattern used in the present study was similar to the three-meal pattern in modern society. Considering the high similarity in anatomy, physiology, polyphagy, habits, metabolism, and gut microbiota between pigs and human beings (15, 16), findings from this study will provide information on the applications of TRF on human beings as well.

## Materials and Methods

### Ethics Statement

This study was approved by the Nanjing Agricultural University Animal Care and Use Committee (Nanjing, Jiangsu Province, China) (SYXK2019-0066). All animal care procedures in the experiment were operated according to the standard of Experimental Animal Care and Use Guidelines of China (EACUGC2018-01).

### Animals, Housing, and Sample

Twelve 105-day growing crossbred barrows (Duroc × Landrace × Large White, average bodyweight = 56.29 ± 1.38 kg) were randomly allotted into two groups with six replicates (1 pig/pen) per group, namely, the free-access feeding group (FA) and TRF group. Pigs in the FA group were fed free-access to feed while the TRF group were fed free-access within a regular time three times per day at 07:00–08:00, 12:00–13:00, and 18:00–19:00, respectively. All pigs were fed with the same commercial pellet feed for growing pigs and *ad libitum* access to water throughout the experiment period. After a 14-day *ad libitum* feeding during the pre-experiment period, the trial started and lasted for 21 days. All pigs were slaughtered and colonic digesta samples were collected on the 21st day. After measuring the pH using a portable pH meter (Hanna Instruments, Villafranca, Italy), all samples were stored under −80°C for microbiota, microbial metabolites, and metabolome analysis.

### DNA Extraction, 16S rRNA Gene Amplification, and Sequencing

Total DNA from colonic digesta was extracted using the cetrimonium bromide (CTAB) method according to a previous method (17). The sequencing was finished by Shanghai Biozeron Biotechnology Co., Ltd (Shanghai, China). Shortly, the V3–V4 regions of the bacterial 16S rRNA gene were amplified using a universal primer with the barcode [forward primer (5′-ACTC CTRCGGGAGGCAGCAG-3′) and a reverse primer (5′-GGACTACCVGGGTATCTAAT-3′)] (18). Then, sequencing libraries were generated using the NEB Next®Ultra™DNA Library Prep Kit for Illumina (NEB, USA) following the manufacturer's recommendations, and index codes were added. At last, the library was sequenced on an Illumina MiSeq platform and 250/300 bp paired-end reads were generated.

QIIME (version 1.17) was used to demultiplex and quality-filter the raw sequence. Reads that were shorter than 50 bp and those that could not be assembled were discarded. Reads with an exact barcode matching and two nucleotide mismatches in primer matching, or containing ambiguous characters, were removed. According to the overlap sequence, only sequences that overlap longer than 10 bp were assembled according to their overlap sequence. Reads which could not be assembled were discarded. Operational taxonomic units (OTUs) were clustered with a 97% similarity cutoff standard using UPARSE (version 7.1, http://drive5.com/uparse/), and chimeric sequences were identified and removed using UCHIME. The phylogenetic affiliation of each 16S rRNA gene sequence was analyzed by the RDP Classifier (http://rdp.cme.msu.edu/) against the silva (SSU115). Rarefaction curve, Shannon and Simpson diversity indices, and Ace and Chao richness estimators were assessed using an online website (MicrobiomeAnalyst, https://www.microbiomeanalyst.ca/). The principal coordinate analysis (PCoA) was conducted based on the Bray–Curtis distance. EdgeR algorithms were used for performing a differential abundance analysis.

### Metabolome Analysis and Data Processing

Metabolome analyses were finished by a commercial company named BioCluster (Shanghai, China). Briefly, 50 mg of the colonic sample was extracted with 800 μL of 80% methanol. A total of 200 μL of supernatant was added with 5 μL of internal standard (140 μg/mL, DL-o-Chlorophenylalanine), then transferred to a vial for LC-MS analysis. LC-MS Analysis was finished using the analysis of the LC-MS platform (Thermo, Ultimate 3000LC, Q Exactive) under the following setups: Column: Hyper gold C18 (100 × 2.1 mm 1.9 μm); Chromatographic separation conditions: Column temperature: 40°C; Flow rate: 0.3 mL/min; Mobile phase A: water +5% acetonitrile +0.1% formic acid; Mobile phase B: acetonitrile +0.1% formic acid; Injection volume: 4 μL; Automatic injector temperature: 4°C; ESI+: Heater Temp 300°C; Sheath Gas Flow rate, 45 arb; Aux Gas Flow Rate, 15 arb; Sweep Gas Flow Rate, 1 arb; spray voltage, 3.0 KV; Capillary Temp, 350°C; S-Lens RF Level, 30%. ESI-: Heater Temp 300°C, Sheath Gas Flow rate, 45 arb; Aux Gas Flow Rate, 15 arb; Sweep Gas Flow Rate, 1 arb; spray voltage, 3.2 KV; Capillary Temp, 350°C; S-Lens RF Level, 60%.

All statistical analyses of the metabolites were done based on the different function modules of a powerful tool, which is available online (version 5.0,) (19). Features with >30% missing values were removed, and the missing values of the remaining features will be replaced by a very small value (half of the minimum positive value found in the data set) by default. No missing values were detected with the criterion. The data then underwent logarithmic transformation and normalization of auto-scaling. Fold change analysis and *T*-test were conducted to determine the fold change and statistical significance of each metabolite from the colonic samples of growing pigs from the TRF group compared with the FA group. PLS-DA was employed to picture the overall difference between the TRF and FA groups, and to explore the differential metabolites. The metabolites with variable importance projection (VIP) values above 1.0, *P* < 0.05 were selected as differential metabolites. The differential metabolites were used to execute an enrichment analysis to explore the main pathway changed by the TRF.

### Short-Chain Fatty Acids (SCFAs)

The concentrations of SCFAs (acetate, propionate, isobutyrate, butyrate, isovalerate, valerate) were measured by the gas chronograph method according to Wang et al. (20). Shortly, 0.25 g of colonic digesta samples was suspended in 1.5 mL distilled water. After vortex-mixing, the suspension was centrifuged at 12,000 × rmp for 10 min at 4°C. A total of 1 mL of the supernatant was taken and mixed with 0.2 mL of 25% (w/v) metaphosphoric acid. The mixture was frozen at 20°C overnight. After thawing, the mixture was centrifuged at 12,000 × rmp for 20 min at 4°C, then 0.5 mL of the supernatant was taken and mixed with an isovolumic ether. Shaking of the mixture was performed to extract the SCFA in the aqueous phase. The organic phase was taken for further analysis using an Agilent 7890A gas chromatograph (Agilent Technologies, Wilmington, DE) equipped with the flame ionization detector (FID), splitless injection port. Standard curves are established using the gradient mixed standards to calculate the concentration of the measured SCFA. The total SCFAs were calculated by adding the six SCFAs measured.

### Lactate

The concentration of lactate was measured by the colorimetric method using the reagent kit (PN: A019-2-1, Nanjing Jiancheng Institute of Biological Engineering, China) following the instructions in the specification.

### Ammonia-N

The concentration of Ammonia-N in the colonic digesta was analyzed by the colorimetric method according to Shen et al. (21). Specifically, 0.1 g of colonic digesta were weighed and suspended in 1.5 mL of 0.2 M HCl. Following vortex-mixing, the suspension was centrifuged at 14,000 × g for 20 min at 4°C. A total of 0.5 mL of the supernatant was taken and mixed with 0.5 mL of 0.08% (w/v) sodium nitroprusside-sodium salicylate and 0.5 mL of sodium hypochlorite-sodium hydroxide solution. The mixture was vortex mixed and left to stand for 10 min. Absorbance at 700 nm was recorded. Gradient ammonium chloride solutions were used to establish a robust standard curve to calculate the concentration of Ammonia-N.

### Biogenic Amines

Biogenic amines including methylamine, tryptamine, putrescine, cadaverine, tyramine, spermidine, and spermine in the colonic digesta were detected by a high-performance liquid chromatography method according to Yang et al. (22). Gradient mixed standards were also analyzed as the samples to establish a robust curve to calculate the concentrations of the biogenic amines.

### Statistical Analysis

The significance of pH, NH4-N, biogenic amines, SCFAs, the differential microbiota, and metabolites between the two groups were analyzed using the unpaired two-tail *t*-test (IBM SPSS Statistics for Windows, Version 21.0). Pearson correlation was analyzed to evaluate the correlation between the differential microorganisms, microbial metabolites, and the features identified from the metabolome. *P* < 0.05 was considered to represent a significant difference. Data visualization was completed using the GraphPad Prism (version 8.0.1, GraphPad Software, San Diego, CA) and R software (version 4.0.4).

## Results

### Bacterial Community Composition in Colonic Digesta of Growing Pigs Under Different Feeding Modes

**Figure 1:**
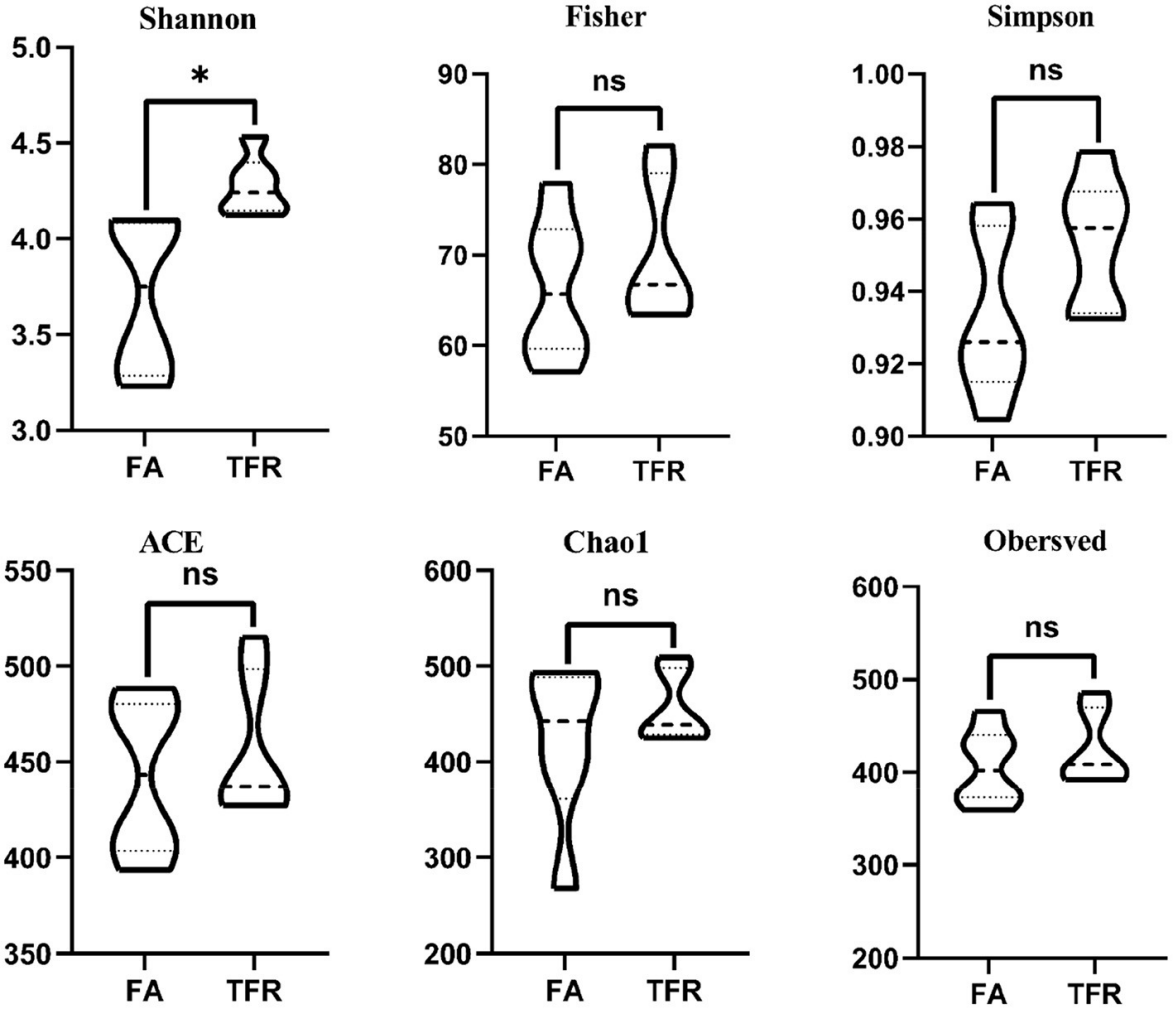
Differences in the colonic bacterial α-diversity index of growing pigs between the FA and TRF groups. FA, free access group; TRF, time-restricted feeding group. *Indicates a significant difference between the two groups; ns represents no significant difference.

**Figure 2:**
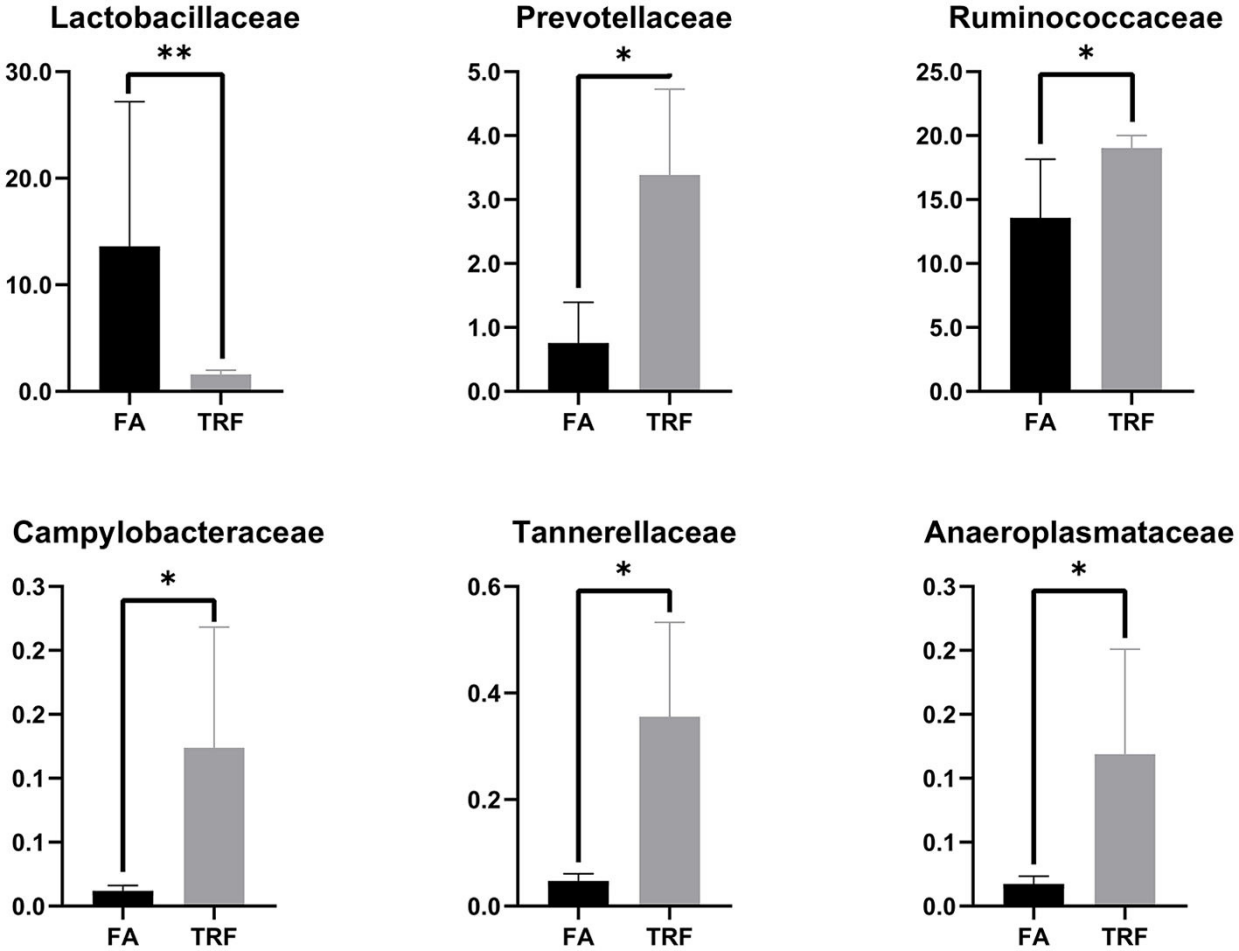
The relative abundance of bacteria in colonic digesta of growing pigs at the family level. FA, free access group; TRF, time-restricted feeding group. **Indicates a significant difference between the FA and TRF groups with *P* < 0.01; while *indicates a significant difference with *P* < 0.05.

**Figure 3:**
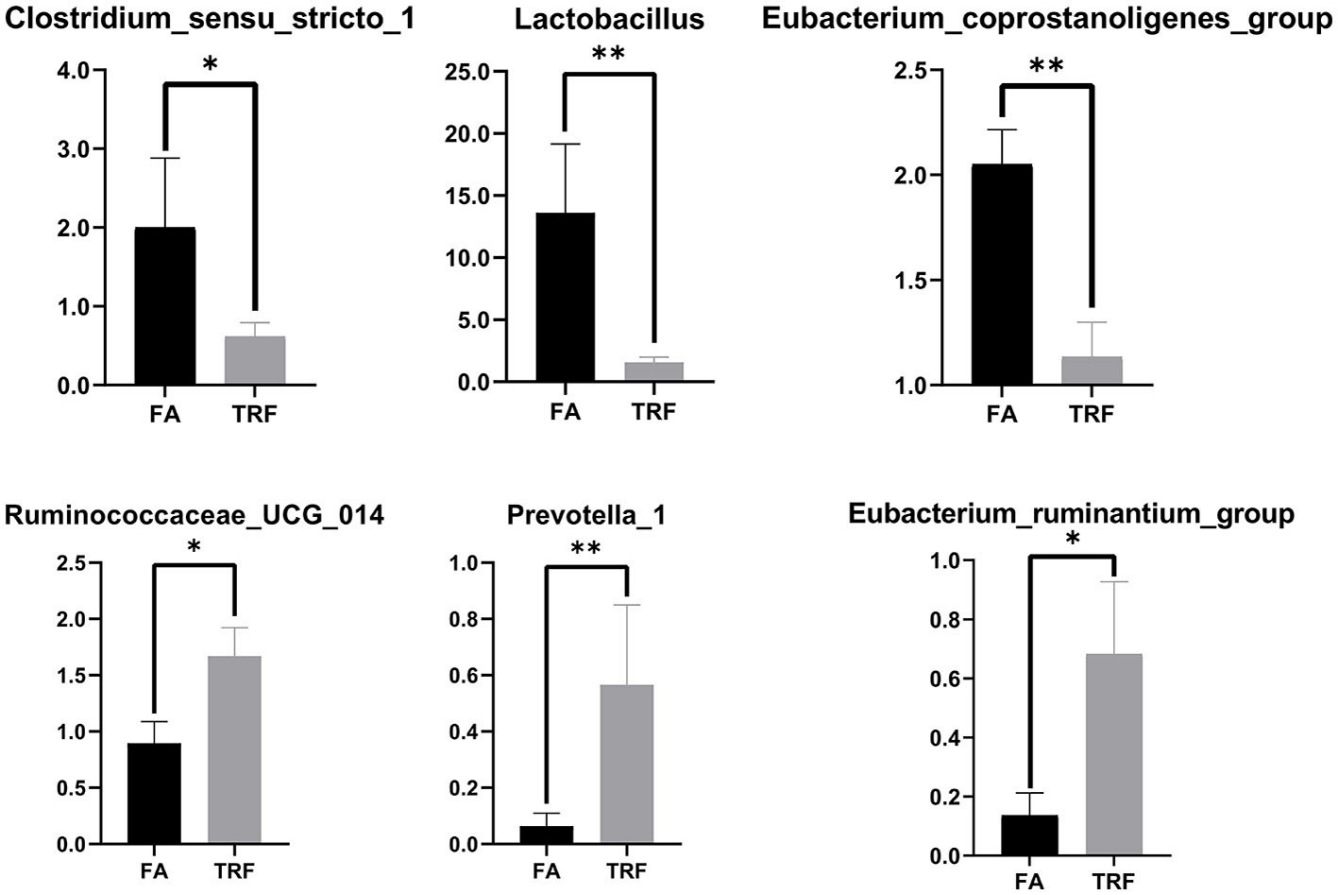
The relative abundances of bacteria in colonic digesta of growing pigs at the genus level. FA, free access group; TRF, time-restricted feeding group. **Indicates a significant difference between the FA and TRF groups with *P* < 0.01; while *indicates a significant difference with *P* < 0.05.

A total of 517,362 clean sequences were obtained after data filtering under a normal quality control system. Using the standard of 97% similarity level, these clean sequences were clustered into 784 OTUs. The rarefaction curves were gradually flattened out with the increasing sampling quantity (Supplementary Figure 1). The α-diversity indexes of the colonic bacterial community were shown in Figure 1. To a large extent, TRF had no significant effects on the Fisher index, Simpson index, ACE index, Chao1 index, and Observed species (*P* > 0.05). However, the Shannon index was significantly higher in the TRF group (*P* = 0.040). Also, the Principal coordinate analysis indicated that TRF could not distinguish the panorama of the colonic bacterial community (Supplementary Figure 2). Whereas, at the family level (Figure 2), the relative abundances of Prevotellaceae (*P* = 0.023), Campylobacteraceae (*P* = 0.010), Tannerellaceae (*P* = 0.012), and Anaeroplasmataceae (*P* = 0.021) were significantly higher in the TRF group compared to those in the FA group, while the relative abundances of Lactobacillaceae (*P* < 0.0014) in the FA group were significantly higher than the TRF group. Meanwhile, at the genus level (Figure 3), the relative abundances of *Prevotella* 1 (*P* = 0.0029) and *Eubacterium ruminantium* group (*P* = 0.017) were significantly higher in the TRF group, and the relative abundances of *Clostridium sensu sticto* 1 (*P* = 0.022), *Lactobacillus* (*P* = 0.002), and *Eubacterium coprostanoligenes group* (*P* = 0.0036) were lower compared with the FA group.

### The pH, Bacterial Metabolites, and the Metabolome in Colonic Digesta of Growing Pigs

**Figure 4:**
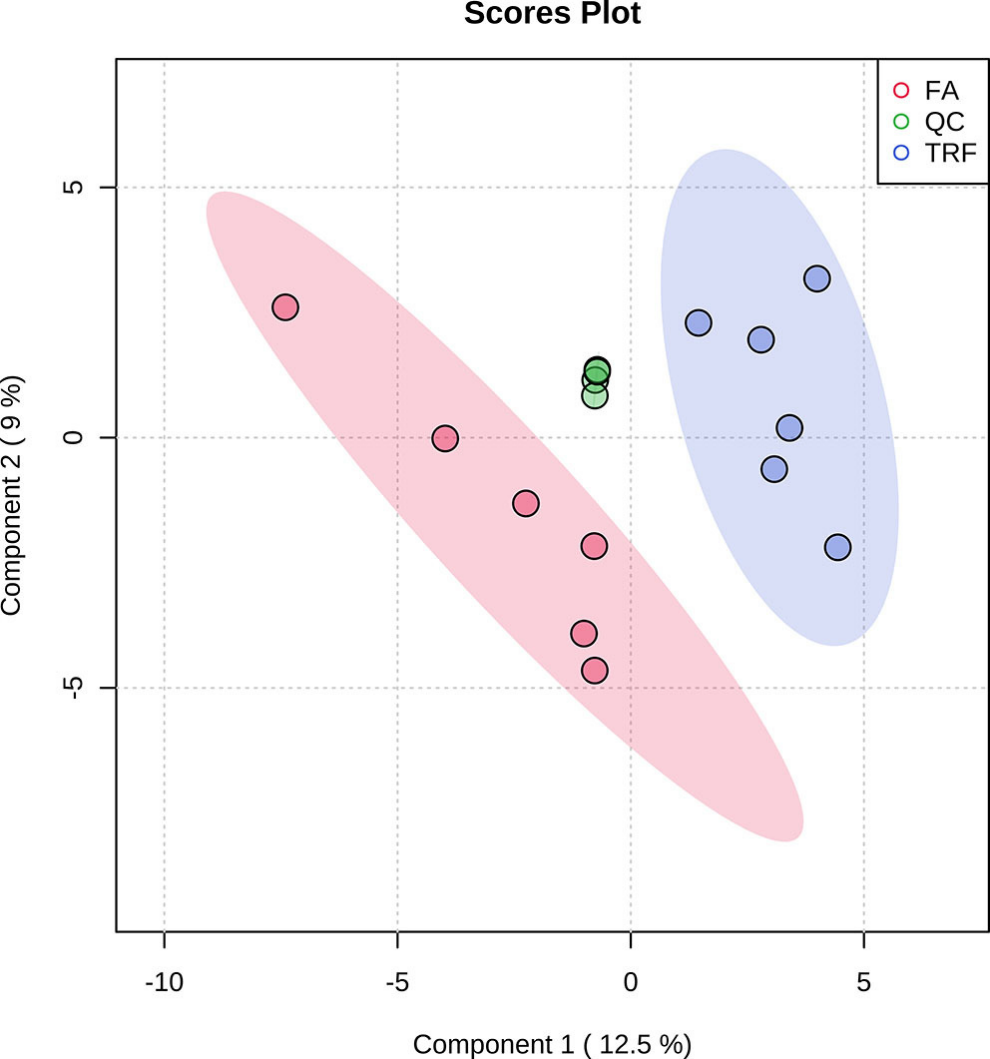
Partial least squares discriminant analysis (PLS-DA) score plot of colonic metabolites (both ESI+ and ESI-) of growing pigs in the FA and TRF groups. FA, free access group; TRF, time-restricted feeding group; QC, quality control. The ellipse represents the 95% confidence interval of each group.

**Figure 5:**
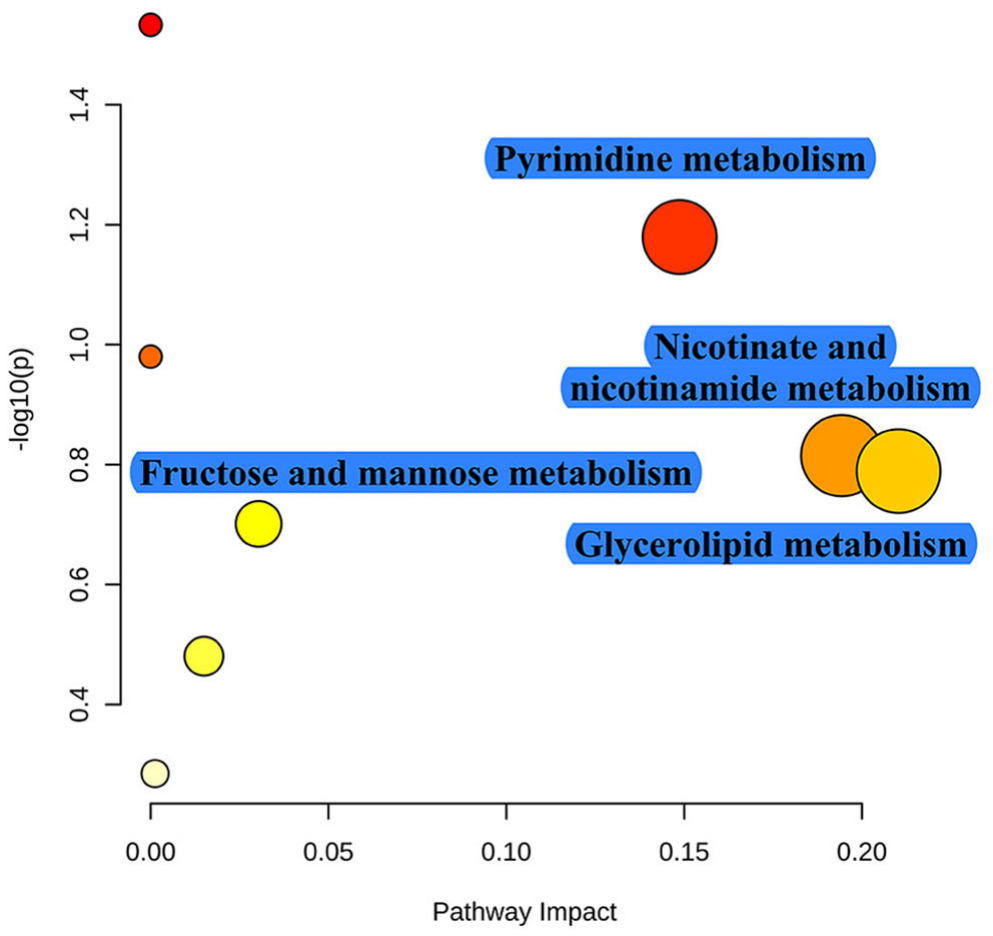
Pathway enrichment of these significant differential metabolites identified between the FA and TRF groups. The x-axis marks the pathway impact and the y-axis represents the pathway enrichment. Each node marks a pathway, with larger sizes and darker colors representing higher pathway enrichment and pathway impact values.

As shown in Table 1, TRF significantly increased the concentrations of NH4-N (*P* < 0.001), putrescine (*P* = 0.003), cadaverine (*P* = 0.010), spermidine (*P* < 0.001), spermine (*P* = 0.014), total biogenic amines (*P* < 0.001), isobutyrate (*P* < 0.001), butyrate (*P* = 0.005), isovalerate (*P* = 0.001), total SCFA (*P* = 0.008), and lactate (*P* = 0.024), while it decreased the pH value and the concentration of acetate (*P* = 0.002) in colonic digesta. TRF had a trend to increase the concentration of methylamine (*P* = 0.083) and tended to decrease the concentration of tyramine (*P* = 0.063). Using the LC-MS metabolomic technique, a total of 258 metabolites in the ESI+ mode and 123 metabolites in the ESI- mode were identified. According to the characteristics of the detected metabolites in each ion mode, one of the repeated metabolites detected in both ESI+ and ESI- modes were eliminated, the remaining 332 metabolites were combined for further analysis. These metabolites mainly included amino acids and their derivatives, organic acids, amines, nucleoside, flavonoid, fatty acids, peptides, etc. As shown in Figure 4, the PLS-DA analysis revealed an obvious and regular variation between the FA and TRF groups. Based on the standards of VIP > 1.0 and *P* < 0.05, a total of 22 significant differential features were identified (Table 2). Results of pathway enrichment analysis showed that these differential features were mainly enriched in pyrimidine metabolism, nicotinate and nicotinamide metabolism, glycerolipid metabolism, and fructose and mannose metabolism (Figure 5).

**Table 1:** The pH value and concentrations of lactic acid, NH4-N, biogenic amines, and SCFAs in colonic digesta of growing pigs.

**Items**	**FA^**a**^**	**TRF^**a**^**	***P***
pH	6.49 ± 0.06	6.26 ± 0.03	0.013
NH4-N (mg/g)	332.79 ± 5.68	376.6 ± 6.78	<0.001
Methylamine (μmol/g)	24.85 ± 3.75	32.2 ± 0.97	0.085
Tryptamine (μmol/g)	75.07 ± 6.26	87.81 ± 6.81	0.163
Putrescine (μmol/g)	49.77 ± 6	75.99 ± 3.01	0.003
Cadaverine (μmol/g)	40.08 ± 3.75	57.44 ± 4.67	0.010
Tyramine (μmol/g)	27.33 ± 4.24	17.44 ± 2.78	0.063
Spermidine (μmol/g)	173.99 ± 14.76	312.13 ± 17.93	<0.001
Spermine (μmol/g)	24.04 ± 4.45	52.61 ± 9.54	0.014
Total biogenic amines (μmol/g)	415.12 ± 19.33	635.62 ± 28.09	<0.001
Acetate (μmol/g)	50.56 ± 0.53	47.11 ± 0.56	0.002
Propionate (μmol/g)	19.67 ± 0.45	19.02 ± 0.45	0.300
Isobutyrate (μmol/g)	2.19 ± 0.11	2.47 ± 0.14	<0.001
Butyrate (μmol/g)	7.38 ± 0.26	8.03 ± 0.15	0.005
Isovalerate (μmol/g)	2.60 ± 0.17	2.99 ± 0.16	0.001
Valerate (μmol/g)	2.36 ± 0.18	2.48 ± 0.2	0.310
Total SCFA (μmol/g)	81.3 ± 0.7	85.55 ± 0.62	0.008
Lactate (μmol/g)	2.20 ± 0.36	3.68 ± 0.5	0.024

a *FA, free access group; TRF, time-restricted feeding group*.

**Table 2:** Identification of significant differential features in colonic digesta of growing pigs.

**NO**.	**Feature**	**FC^**a**^**	**log_**2**_(FC)**	***P***	**VIP^**b**^**	**Mode**
1	Hexadecanedioic acid	0.53477	−0.90302	<0.001	1.2162	ESI-
2	dTMP	2.9032	1.5376	0.001	1.2006	ESI-
3	Glycylleucine	0.55638	−0.84587	0.002	1.1357	ESI-
4	Methyl-beta-galactopyranoside	0.36016	−1.4733	0.002	1.0248	ESI-
5	Fructose 1-phosphate	7.7772	2.9593	0.003	1.0098	ESI-
6	Hippuric acid	0.43986	−1.1849	0.003	2.416	ESI-
7	Urobilin	0.53018	−0.91544	0.004	1.0603	ESI-
8	16-Hydroxyhexadecanoic acid	0.65938	−0.60082	0.010	1.1402	ESI-
9	Thymidine	1.5709	0.65161	0.031	1.5147	ESI-
10	Lysophosphatidylethanolamine	1.6385	0.71238	0.041	1.3816	ESI-
11	Serinyl-Leucine	0.52303	−0.93502	0.002	1.0933	ESI+
12	Thymidine	1.7536	0.81031	0.003	1.2584	ESI+
13	Adenosine	1.7304	0.79107	0.006	1.0149	ESI+
14	1-Methyladenosine	2.0063	1.0046	0.009	1.1544	ESI+
15	Monoglyceride	1.9224	0.94294	0.009	2.0194	ESI+
16	D-2-Aminobutyric acid	0.5904	−0.76023	0.011	1.9605	ESI+
17	Asparaginyl-glutamic acid	1.6256	0.70102	0.027	1.2668	ESI+
18	DL-pipecolic acid	2.8771	1.5246	0.030	1.52	ESI+
19	N6, N6, N6-Trimethyl-L-lysine	1.8283	0.87054	0.039	1.4042	ESI+
20	Niacinamide	0.53713	−0.89665	0.039	1.4031	ESI+
21	Cytidine	1.6513	0.72356	0.042	1.3731	ESI+
22	Dihydroartemisinin	1.8634	0.89791	0.043	1.3595	ESI+

a *FC, fold change*;

b *VIP, variable importance in the projection*.

### Correlations Between the Bacterial Genera, Bacterial Metabolites, and Differential Features in Colonic Digesta of Growing Pigs

**Figure 6:**
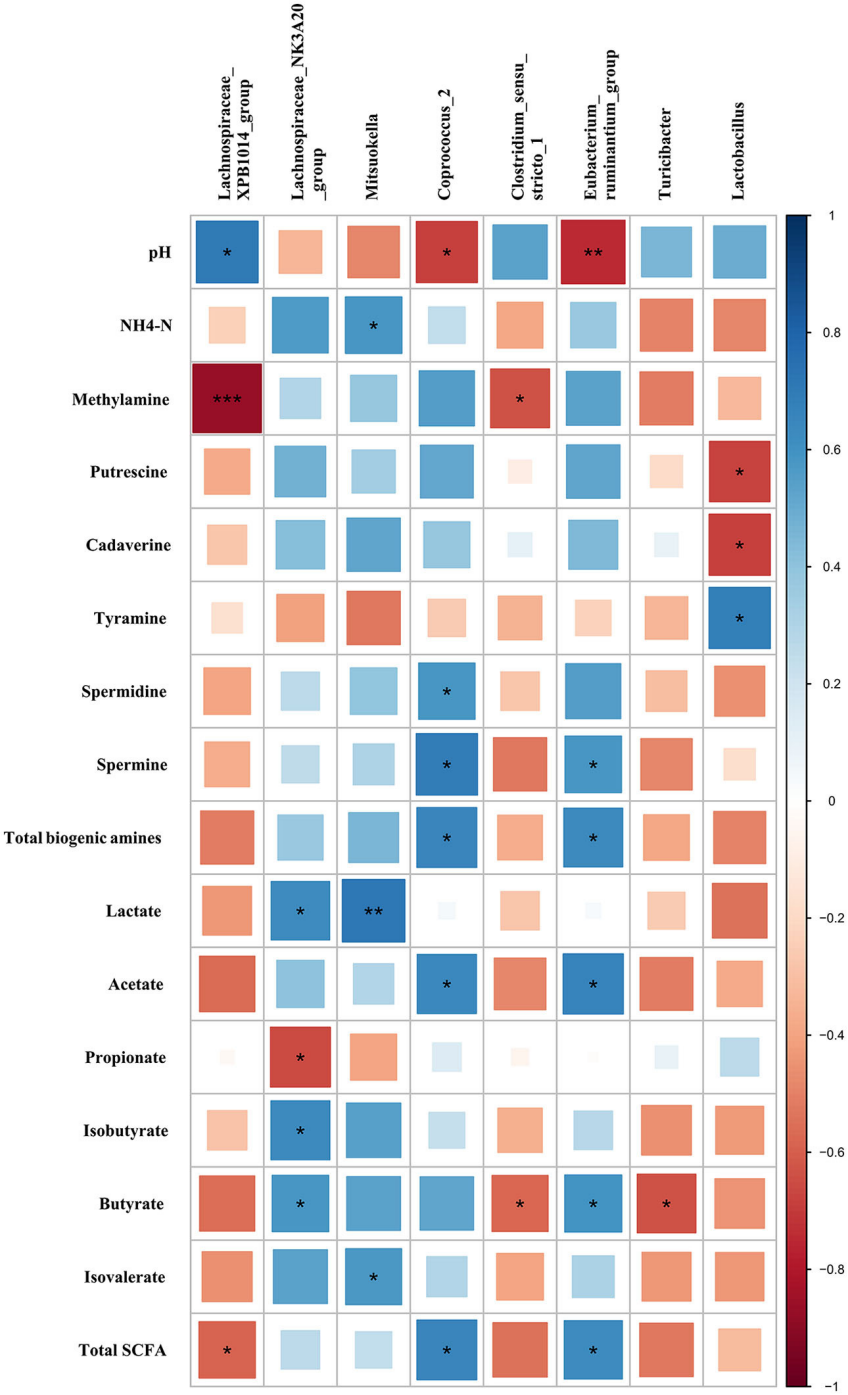
Correlations between the differential microbiota at the genus level with bacterial metabolites in colonic digesta of growing pigs. Each row in the graph represents a pH value and bacterial metabolite (including SCFAs, biogenic amines, lactate, and NH4-N), each column represents a genus, each square represents a Pearson correlation coefficient between a genus and a bacterial metabolite (or pH), while the area of each square represents the size of each correlation coefficient. Red color represents a negative correlation, while blue color represents a positive correlation. ***Indicates a significant difference between the free access group and time-restricted feeding group with *P* < 0.001; while **, *indicates significant difference with *P* < 0.01, and *P* < 0.05 respectively.

Based on the Pearson correlation analysis, the differential microbiota at the genus level showed a significant correlation with bacterial metabolites in colonic digesta (Figure 6). Specifically, *Lachnospiraceae XPB1014 group* was negatively correlated with methylamine (*r* = −0.86, *P* < 0.001) and total SCFA (*r* = −0.58, *P* = 0.047), while it was positively correlated with pH (*r* = 0.70, *P* = 0.01). *Lachnospiraceae NK3A20 group* was negatively correlated with propionate (*r* = −0.66, *P* = 0.020), while it was positively correlated with lactate (*r* = 0.63, *P* = 0.029), isobutyrate (*r* = 0.64, *P* = 0.025), and butyrate (*r* = 0.58, *P* = 0.046). *Mitsuokella* was positively correlated with NH4-N (*r* = 0.59, *P* = 0.045), lactate (*r* = 0.72, *P* = 0.0082), and isovalerate (*r* = 0.58, *P* = 0.049). *Coprococcus 2* was negatively correlated with pH (*r* = −0.69, *P* = 0.010), while it was positively correlated with *spermidine* (*r* = 0.58, *P* = 0.046), spermine (*r* = 0.69, *P* = 0.012), total biogenic amines (*r* = 0.66, *P* = 0.020), acetate (*r* = 0.64, *P* = 0.024), and total SCFA (*r* = 0.66, *P* = 0.020). *Clostridium sensu stricto 1* was negatively correlated with methylamine (*r* = −0.64, *P* = 0.026) and butyrate (*r* = −0.58, *P* = 0.049). *Eubacterium ruminantium group* was negatively correlated with pH (*r* = −0.74, *P* = 0.0055), while it was positively correlated with spermine (*r* = 0.59, *P* = 0.045), total biogenic amines (*r* = 0.64, *P* = 0.026), acetate (*r* = 0.66, *P* = 0.018), butyrate (*r* = 0.60, *P* = 0.040), and total SCFA (*r* = 0.62, *P* = 0.030). *Turicibacter* was negatively correlated with butyrate (*r* = −0.63, *P* = 0.028). *Lactobacillus* was negatively correlated with putrescine (*r* = −0.67, *P* = 0.017) and cadaverine (*r* = −0.69, *P* = 0.013), while it was positively correlated with tyramine (*r* = 0.69, *P* = 0.013).

**Figure 7:**
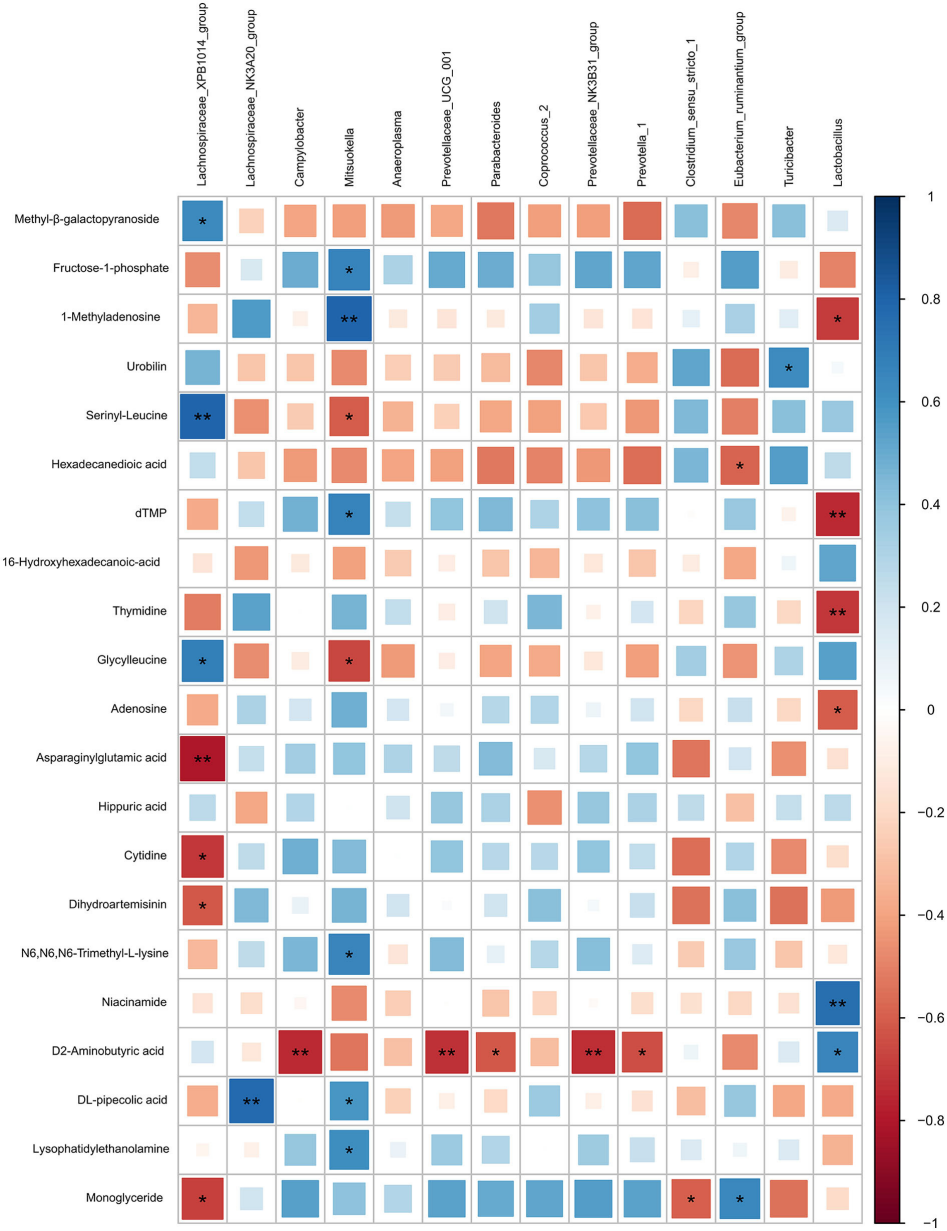
Correlation analysis between the differential microbiota at the genus level with the differential features identified from the LC-MS metabolome in colonic digesta of growing pigs. Each row in the graph represents the differential features, each column represents a genus, each square represents a Pearson correlation coefficient between a genus and a differential feature, while the area of each square represents the size of each correlation coefficient. Red color represents a negative correlation, while blue color represents a positive correlation. **Indicates a significant difference between the free access group and the time-restricted feeding group with *P* < 0.01; while *indicates a significant difference with *P* < 0.05.

Based on the Pearson correlation analysis, the differential microbiota at the genus level showed a significant correlation with the significant differential features in colonic digesta (Figure 7). Specifically, *Lachnospiraceae XPB1014* group was negatively correlated with cytidine (*r* = −0.70, *P* = 0.011), dihydroartemisinin (*r* = −0.61, *P* = 0.033), asparaginylglutamic acid (*r* = −0.80, *P* = 0.017), and monoglyceride (*r* = −0.69, *P* = 0.014), while it was positively correlated with serinyl-leucine (*r* = 0.80, *P* = 0.0019), glycylleucine (*r* = 0.68, *P* = 0.014), and methyl-β-galactopyranoside (*r* = 0.64, *P* = 0.025). *Lachnospiraceae NK3A20* group was positively correlated with DL-pipecolic acid (*r* = 0.78, *P* = 0.0029). *Campylobacter* (*r* = −0.74, *P* = 0.0056), *Prevotellaceae NK3B31 group* (*r* = −0.74, *P* = 0.0060), *Parabacteroides* (*r* = −0.61, *P* = 0.0034), *Prevotella 1* (*r* = −0.64, *P* = 0.0024), and *Prevotellaceae UCG 001* (*r* = −0.73, *P* = 0.0070) were negatively correlated with D-2-aminobutyric acid. *Mitsuokella* was positively correlated with 1-methyladenosine (*r* = 0.79, *P* = 0.0020), N6,N6,N6-trimethyl-L-lysine (*r* = 0.66, *P* = 0.020), DL-pipecolic acid (*r* = 0.58, *P* = 0.047), dTMP (*r* = 0.67, *P* = 0.018), fructose-1-phosphate (*r* = 0.67, *P* = 0.018), and Lysophosphatidylethanolamine (*r* = 0.61, *P* = 0.034), while it was negatively correlated with serinyl-leucine (*r* = −0.60, *P* = 0.038) and glycylleucine (*r* = −0.67, *P* = 0.018). Turicibacter was positively correlated with urobilin (*r* = 0.62, *P* = 0.030). *Clostridium sensu stricto 1* was negatively correlated with monoglyceride (*r* = −0.60, *P* = 0.040). *Eubacterium ruminantium group* was positively correlated with monoglyceride (*r* = 0.64, *P* = 0.024), while it was negatively correlated with hexadecanedioic acid (*r* = −0.58, *P* = 0.046). *Lactobacillus* was negatively correlated with 1-methyladenosine (*r* = −0.70, *P* = 0.011), adenosine (*r* = −0.61, *P* = 0.036), thymidine (*r* = −0.71, *P* = 0.010), and dTMP (*r* = −0.74, *P* = 0.0056), while it was positively correlated with niacinamide (*r* = 0.76, *P* = 0.004) and D-2-aminobutyric acid (*r* = 0.65, *P* = 0.02).

## Discussion

Growing evidence suggests that the nutrients (including fatty acids, amino acids, and saccharides) and the luminal environment (e.g., pH) shapes the microbiota community (23–25). In the present study, the overall composition of the colonic microbiota communities was not changed between the two groups, the most likely reason is that all pigs in both groups were fed with the same commercial feed. However, the Shannon index was significantly increased in the present study indicating that the TRF group improved the evenness of the microbial community. As reported, metabolite cross-feeding promotes a suboptimal community growth and shapes the species diversity in the gut microbiota (26). Therefore, the results may be contributed to the nutritional variations in the colonic lumen along with the different feeding rhythms. Under the free-access mode, a growing pig eats about seven times per day with an average feed intake of 260 g (27). However, our research found that the phased feed intake of growing pigs in a day underwent diurnal rhythmicity and peaked at 12:00–16:00 (unpublished data), which means that a fluctuation in nutrients may exist in the colonic lumen. By contrast, pigs in the TRF group were fed access to feed at identical intervals which may lead to a relative evenness of the nutrients, thus, affecting the microbiota community meanwhile.

A plethora of studies has reported that the bidirectional interactions between the host and the intestinal microbiota are tightly regulated to maintain health and homeostasis (28, 29). Gut microbiota plays pivotal roles in maintaining metabolic hemostasis including the metabolism of amino acids, carbohydrates, lipid, nucleotide, and vitamins (29). Vice versa, nutrients in the intestine taking by the host could domesticate the gut microbes meanwhile (28). Although the overall microbial composition was not considerably different, the TRF group significantly altered the relative abundance of certain taxa like *Lactobacillus* at the genus level. Surprisingly, we found that the TRF group has decreased the relative abundance of *Lactobacillus*, however, the decrease did not reduce the content of lactate. These results were inconsistent with those reported previously (30, 31). Zhang et al. (32) have reported that the oral administration of the *Lactobacillus* strain has increased the production of lactate. The following reasons may explain these results. Firstly, *Bifidobacterium* and *Enterococcus*, as well as *Lactobacillus*, could produce lactate (33). Thus, in line with Zhang et al. (32), we found that the relative abundance of *Lactobacillus* was not directly correlated with the concentration of lactate. Secondly, as a secondary metabolite, the lactate produced was possibly consumed simultaneously by yeasts or other aerobic bacteria in the FA group (34). Besides, the production efficiency of lactate during fermentation by different strains of *Lactobacillus* was not identical, especially with different substrates (35). Researchers have found that *Mitsuokella* had a positive effect on the serum free amino acids in weaned piglets (32), while in the present study, we found that *Mitsuokella* had a negative correlation with serinyl-leucine and glycylleucine. *Eubacterium coprostanoligenes* group has been reported to have a function of bio-transforming cholesterol to coprostanol which could further influence the fat metabolism of the host (36). Cholesterol has been shown to exert crucial physiological effects on animals (37). While in the present study, TRF has significantly reduced the relative abundance of *Eubacterium coprostanoligenes* group which possibly indicates that more cholesterol was utilized in the TRF group. Consistently, differential metabolites were enriched in fat-related metabolism pathways. *Prevotella* has been reported to associate with the increase of host feed intake (38). However, the feed intake was not affected by TRF in the present study (unpublished data). The possible reason was that although the relative abundance of Prevotellaceae at the family level was increased by TRF, it did not change the relative abundance of Prevotella at the genus level, but increased the relative abundance of Prevotella 1. Despite relating to several diseases (39), several members of Prevotellaceae were reported to produce succinate and could improve the glucose homeostasis status by activating intestinal gluconeogenesis (40). Thus, through promoting host health or energy metabolism, Prevotellaceae was reported to increase the feed efficacy in pigs (41). Herein, we found that TRF has significantly increased the relative abundance of Prevotellaceae at the family level which suggests that TRF would probably improve the production efficacy of swine production. As an evidence, the FCR was indeed improved by TRF in the present study (unpublished data).

Microbial metabolic products, mainly including SCFAs and biogenic amines, are thought to mediate the beneficial health effects of the intestinal microbial community (42). SCFAs were the main end-products of microorganisms fermenting carbohydrates, which are generally believed to have a benefit to host health (43). Specifically, butyrate, as one of the most important SCFAs, has extensive effects on improving immunity and intestinal health and promoting host metabolism (3, 44). In the present study, we found that TRF has increased the concentration of butyrate which indirectly reflected that TRF could improve host metabolism. Accordingly, the *Lachnospiraceae NK3A20* group, which was positively correlated with butyrate, was found to be increased by TRF treatment (45). Besides, we also found that D2-aminobutyric acid, an unnatural chiral α-amino acid, in colonic digesta of growing pigs had excessive correlations with the differential bacterial genera. Unfortunately, there are currently few studies concerning the physiological function of D2-aminobutyric acid, thus, further research is still needed. Biogenic amines are produced by intestinal microbiota through the decarboxylation of aromatic or cationic amino acids (46). In the present study, we found that the TRF mode had a trend to decrease the concentration of tyramine. Furthermore, we found that *Lactobacillus* had a tight correlation with tyramine. Research has found that *Lactobacillus* can deaminate proteins (47). Moreover, researchers have found that 28% of the *Lactobacillus* produced tyramine (48). In the present study, TRF has increased the concentration of putrescine, spermidine, and spermine. Putrescine was reported to stimulate the synthesis of epithelial DNA and RNA (49). In line with this notion, pathway analysis indicates that TRF has increased the progress of pyrimidine metabolism. Furthermore, putrescine could mitigate intestinal atrophy by suppressing inflammatory responses in weanling piglets (50). Spermidine and spermine were reported to promote adipogenesis (51, 52), while cadaverine exerts protective effects on epithelial cells (53). Consistently, researchers have found that *Lactobacillus* was negatively correlated with cadaverine (25). The decreased production of biogenic amines may be contributed to the inhibitory effects of *Lactobacillus* on the growth of amine-positive bacteria (54). However, some genera of amine positive lactic acid bacteria strains, including *Lactobacillus plantarum* (FI8595) and *Lactococcus lactis subsp. cremoris* (MG 1363), had a crucial role on the increase of cadaverine and accumulation of putrescine (55). However, the biogenic amines function probably depends on the dose and physiological state of the host (56, 57).

## Conclusions

In conclusion, the overall composition of gut microbiota community between the two groups was not influenced. However, TRF treatment significantly altered the relative abundance of certain families and genera including *Lactobacillus, Eubacterium ruminantium* group, *Eubacterium coprostanoligenes* group, *Prevotella* 1, and *Clostridium sensu sticto* 1. Furthermore, TRF treatment induced the gradient changes of metabolites in colonic digesta. Interestingly, the correlation results suggested that gradients of metabolites induced by TRF were correlated with the differential microbial genera indicating that the metabolites might mediate the effect of TFR on the gut microbiota. However, more research is needed to understand the benefits and risks of TRF on the health and metabolism of growing pigs.

## Data Availability Statement

The data presented in the study are deposited in the repository of Sequence Read Archive, accession number: PRJNA715648.

## Ethics Statement

The animal study was reviewed and approved by Nanjing Agricultural University Animal Care and Use Committee (Nanjing, Jiangsu Province, China) (SYXK2019-0066).

## Author Contributions

YS and WZ conceived and designed the experiments. HW, PX, ZL, and YS performed the experiments and analyzed the data. HW and YS wrote the paper. All authors contributed to the article and approved the submitted version.

## Conflict of Interest

The authors declare that the research was conducted in the absence of any commercial or financial relationships that could be construed as a potential conflict of interest.

## Supplementary Material

Supplementary Figure 1 Rarefaction curve comparing the number of OTUs found in the 16S rDNA gene libraries from microbiota in the colon.

Supplementary Figure 2 Principal coordinate analysis (PcoA) of the colonic bacterial community of the growing pigs (*n* = 6). FA, free access group; TRF, time-restricted feeding group.
