# Supplementary material for: Metabolome-Microbiome Responses of Growing Pigs Induced by Time-Restricted Feeding
Source: Front Vet Sci. 2021 Jun 22;8:681202. doi: 10.3389/fvets.2021.681202 (PMC8258120; doi:10.3389/fvets.2021.681202)
Supplement: Supplementary Figure 1 — Rarefaction curve comparing the number of OTUs found in the 16S rDNA gene libraries from microbiota in the colon. [file Data_Sheet_1.PDF]

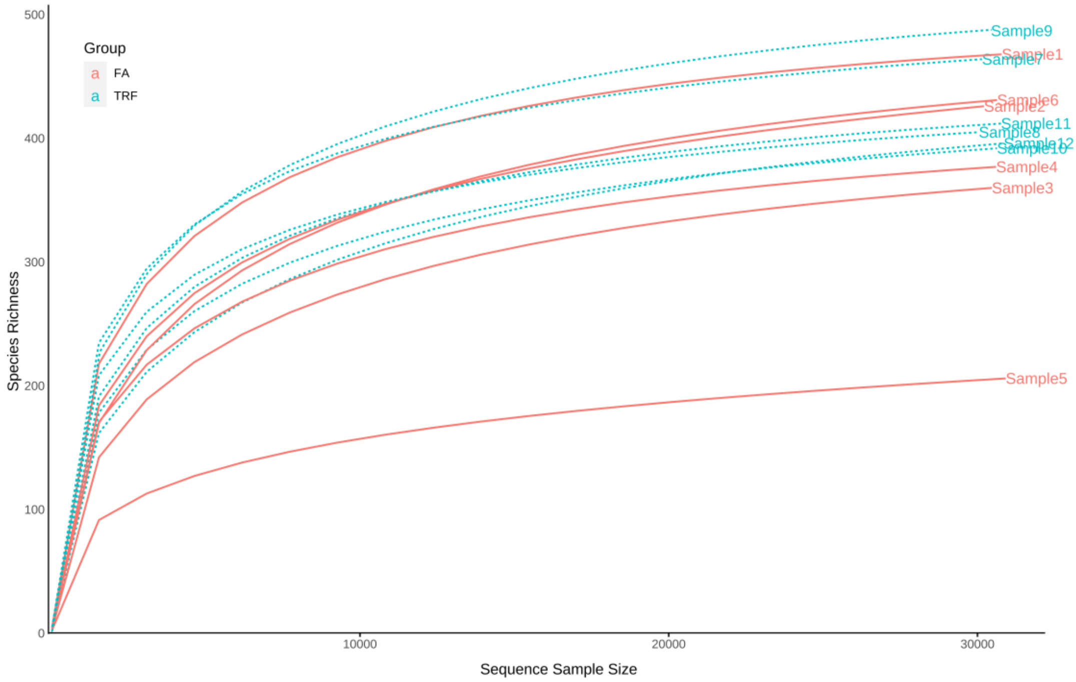

Figure S1 Rarefaction curve comparing the number of OTUs found in the 16S rDNA gene libraries from microbiota in the colon.

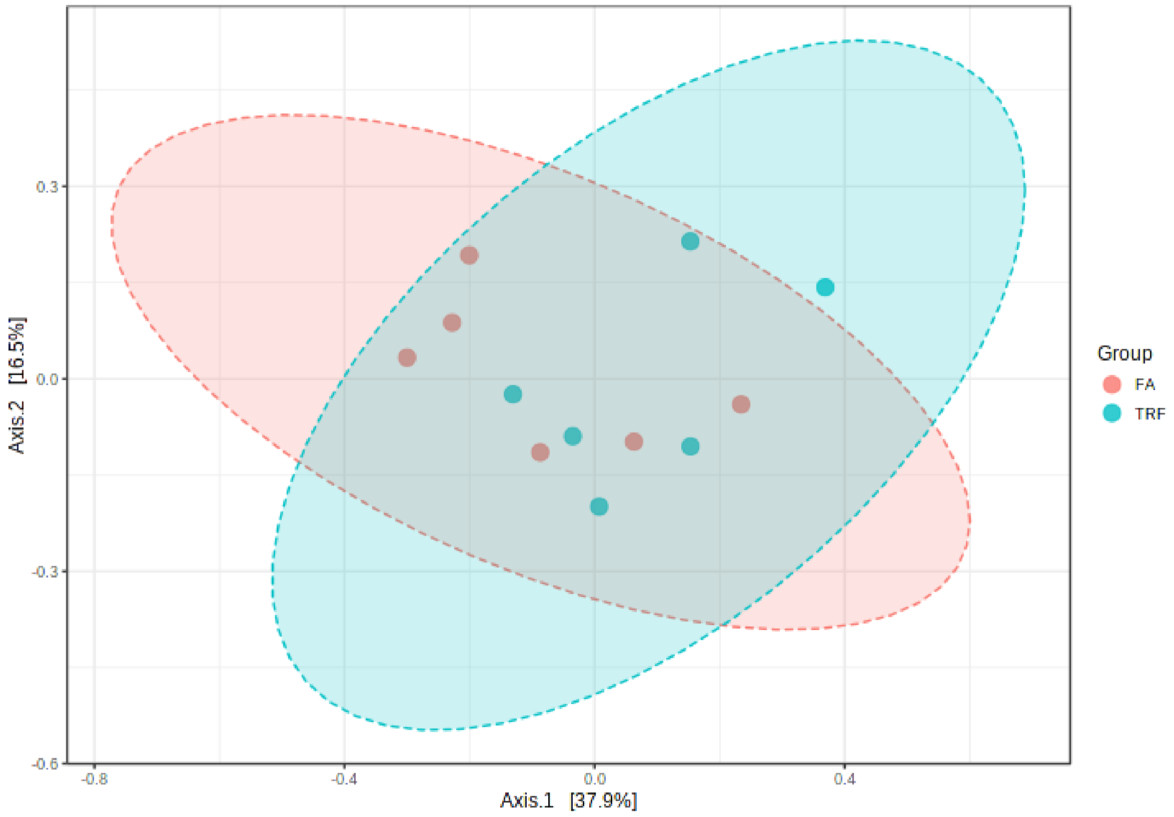

Figure S2 Principal coordinate analysis (PcoA) of the colonic bacterial community of the growing pigs (n=6). FA, free access group; TRF, time-restricted feeding group.
